# Does the experience of the first assistant affect organ injuries in laparoscopic hysterectomy for benign diseases?

**DOI:** 10.1007/s00404-022-06745-4

**Published:** 2022-09-01

**Authors:** Yoko Tsuzuki, Takumi Hirata, Shinya Tsuzuki, Shinichiro Wada, Akiko Tamakoshi

**Affiliations:** 1 Department of Obstetrics and Gynecology, Teine Keijinkai Hospital, 1-40, 1-jou, 12-choume, Maeda, Teine-ku, Sapporo city, Hokkaido, 006-8555 Japan; 2 Department of Public Health, Hokkaido University Faculty and Graduate School of Medicine, Kita 15, Nishi 7, Kita-ku, Sapporo city, Hokkaido, 060-8638 Japan; 3 Disease Control and Prevention Center, National Center for Global Health and Medicine, 1-21-1, Toyama, Shinjuku-ku, Tokyo, 162-8655 Japan

**Keywords:** Assistant, Complication, Hysterectomy, Laparoscopy, Organ injury

## Abstract

**Purpose:**

This study sought to explore whether the experience level of the first assistant surgeon influences perioperative organ injuries (ureteral, bladder, and intestinal injuries) in patients undergoing total laparoscopic hysterectomy (TLH) for benign diseases. We defined an experienced surgeon as a surgeon certified by the Skill Qualification Committee of the Japan Society of Gynecologic and Obstetric Endoscopy and Minimally Invasive Therapy or a surgeon with equivalent surgical skills.

**Methods:**

We reviewed our surgical registry database of TLH for benign indications between 2014 and 2020 and only selected cases performed by an experienced primary surgeon. Patients were divided into two groups based on the experience level of the first assistant. Inverse probability of treatment weighting by propensity score, which was adjusted for patient and procedure characteristics, was used to examine differences in perioperative organ injuries according to the experience level of the first assistant.

**Results:**

Among 1682 patients who underwent TLH, 18 organ injuries were found (0.83%). In the propensity score inverse probability of treatment weighting models, less experience of the first assistant had no significant impact on the occurrence of perioperative organ injuries (*p* = 0.348).

**Conclusion:**

In TLH for benign indications at our hospital, given an experienced primary surgeon, the inclusion of a less experienced first assistant does not negatively affect the occurrence of perioperative organ injuries.

**Supplementary Information:**

The online version contains supplementary material available at 10.1007/s00404-022-06745-4.

## What does this study add to the clinical work

**Table:** 

It is recommended that inexperienced first assistants participate in TLH for training and experience while considering the difficulty of the surgical cases.	

## Introduction

Throughout the last three decades, laparoscopic surgery has replaced laparotomy for gynecological surgery [1–3]. Hysterectomy is one of the most common gynecologic surgical procedures worldwide, especially for benign conditions [4, 5]. Compared with open surgery, laparoscopic surgery for hysterectomy is regarded as a minimally invasive approach on the basis of decreased blood loss, length of hospital stay, postoperative pain, risk of infection, and shorter overall recovery time [6–8]. Because of the obvious advantages of total laparoscopic hysterectomy (TLH), it is imperative that gynecologists receive adequate exposure to this technique to gain experience [9]. Compared with abdominal and vaginal hysterectomies, TLH requires greater surgical expertise [7], a longer time to master, and a longer operating time [7, 10]. As surgeons perform more laparoscopic procedures, it is prudent to consider how many operations an individual surgeon must perform to be considered competent [11]. The number of cases needed to achieve an experienced level of performance of TLH has been reported to range from 21 to 75 [4, 12, 13].

However, primary surgeons cannot successfully perform laparoscopic surgery alone and inevitably require a surgical team that includes assistant surgeons [14]. At our institution, TLH is aided by two assistants. The first assistant holds the laparoscope, while the second assistant moves the uterine manipulator.

In a teaching hospital such as ours, trainees including novices in laparoscopic surgery and residents are continuously rotated for surgical training purposes. The experience of the first assistant is essential for trainees of TLH so that they can acquire the skills of a primary surgeon. Assistants understand the details of the operative procedure, learn effective methods to support the operator, and ensure that their experience is useful for their own future performance of the procedure.

Though TLH has many advantages mentioned above, when serious complications, especially injuries in organs adjacent to the uterus (ureteral, bladder, and intestinal injuries), occur during the surgery, conversion to laparotomy is frequently needed [15], which increases the secondary burden and stress on patients [16].

To find a balance between patient safety and trainee education, the effects on the occurrence of severe complications must be scrutinized. Previous investigations have highlighted the relationship between the experience of primary surgeons and perioperative complications in TLH [9, 17–19]. However, to our knowledge, little is known about the impact of the first assistant surgeon’s experience in TLH on the development of complications. Therefore, in the current study, we aim to identify the influence of the first assistant surgeon’s experience on the occurrence of perioperative organ injuries (ureteral, bladder, and intestinal injuries) in TLH for benign diseases.

## Methods

### Study design and population

We conducted a retrospective cohort study of 1,682 women who underwent TLH for benign indications between January 1, 2014 and December 31, 2020 at Teine Keijinkai Hospital in Sapporo, Japan. All TLH procedures were performed by fully qualified and experienced primary surgeons. All patients were admitted to the hospital one day before surgery, and they received standard prophylactic antibiotics. A blood test was performed on postoperative day (POD) 2 according to our department’s clinical protocol (Table S1).

### Exposure measurements

Data on 12 variables were collected (Table S2), and they included demographic data, comorbidities, preoperative use of gonadotropin-releasing hormone (GnRH) agonists, surgical data, surgical techniques in addition to TLH, and diagnosis. We defined an experienced surgeon, including both the operator and the first assistant, as a surgeon certified by the Skill Qualification Committee of the Japan Society of Gynecologic and Obstetric Endoscopy and Minimally Invasive Therapy or a surgeon with equivalent surgical skills. A surgeon with equivalent surgical skills to those of the certified endoscopic surgeon was defined as a surgeon familiar with TLH who had performed at least 75 TLH surgeries in the past, as mentioned above [4, 12, 13]. To acquire this qualification, applicants are required to perform over 100 gynecologic laparoscopic surgeries over a 2-year period after qualification as a specialist recognized by the Japan Society of Obstetrics and Gynecology; they are also required to pass an examination involving a video of the surgery. We believed that this qualification could only be obtained by a surgeon whose skills had improved after appropriate training and that a technically certified surgeon could be considered an experienced surgeon. Because our hospital is a teaching hospital, many young physicians leave within 6 months to a few years. Therefore, by the aforementioned definition, none of the inexperienced surgeons trained at our hospital became experienced surgeons.

### Outcome measurements

To identify patients with organ injuries (ureteral, bladder, and intestinal injuries) related to TLH, medical records were evaluated within 90 days of TLH. Evidence of perioperative organ injuries was determined by review of the operative report, imaging, or a urology and surgery consultation note.

### Statistical analysis

Data were reported as medians (interquartile ranges [IQRs]) for continuous variables. Categorical variables were depicted as absolute numbers and proportions and reported as numbers (%). We divided patients into two groups according to whether an experienced first assistant was present during surgery. To identify the risk factors associated with organ injuries during the 90 days after TLH, univariate analyses were performed. To compare each variable between the two groups, the chi-squared test or Fisher’s exact test was used for categorical variables, and the Mann–Whitney *U* test was used for continuous variables because all continuous variables follow nonparametric distributions.

Propensity score (PS)-based inverse probability of treatment weighting (IPTW) was fitted to confirm the background differences in the two groups in assessing whether the presence or absence of an experienced first assistant affected the morbidity rate of organ injuries. First, the PS for the group with an experienced first assistant was determined by fitting a binary logistic regression model. Patient demographics, comorbidities, preoperative use of a GnRH agonist, surgical data, surgical techniques in addition to TLH, and diagnoses were entered into the final model. Then, an IPTW approach was used to assign the group with an experienced first assistant a weight of 1/PS, and the group without an experienced first assistant, a weight of 1/(1 − PS). The cases in which the PS values were greater than 0.1 were included in the analysis. In the PS-IPTW model, proportional distribution of baseline covariates was assessed by the standardized mean difference (SMD). The two groups were considered to be well balanced when the SMD was less than 0.1. We tested for the differences in organ injury rate between the two groups using Poisson regression. Two-sided *p* values < 0.05 were considered to show statistical significance. We used the statistical software R 3.6.1 for the statistical analyses [20].

### Ethics

This study was approved by the research ethics committee of Teine Keijinkai Hospital (approval no.: 2-020337-00). Informed consent was obtained in the form of opt-out on the website of Teine Keijinkai Hospital.

## Results

Of the 1,682 patients, 14 (0.83%) developed organ injuries (ureteral, bladder, and intestinal injuries) during the 90 days after TLH. Five (35.7%) had ureteral injuries, two (14.3%) had bladder injuries, six (42.9%) had intestinal injuries, and one (7.1%) had both bladder and intestinal injuries (Table 1). The result of univariate analysis and baseline characteristics of patients before and after inverse probability weighting in this study are presented in Table 2. In the univariate analysis, in the group with an experienced first assistant, the rates of diabetes mellitus (*p* value ≤ 0.001) and prior pelvic surgery (*p* value = 0.038) were significantly lower, whereas the rate of preoperative use of a GnRH agonist was significantly higher (*p* value ≤ 0.001) than that in the group without an experienced first assistant.

**Table 1:** List of cases according to characteristics of organ injuries, time to recognize injuries, and subsequent injury management

Case No	Type of organ injury	Date of diagnosis	Surgical technique	With an experienced first assistant	Management
1	Ureteral	During surgery	TLH^2^ + LBS^3^	Yes	Intraoperative abdominal ureterocystoneostomy
2	Ureteral	During surgery	TLH + LBS	Yes	Intraoperative abdominal ureteroureterostomy
3	Ureteral	During surgery	TLH + LLSO^4^ + LRC^5^ + LRS^6^	No	Intraoperative abdominal ureteroureterostomy
4	Ureteral	POD^1^ 6	TLH + LLSO + LRS	Yes	Abdominal ureterocystoneostomy on POD 14
5	Ureteral	POD 11	TLH	No	Ureteral stent placement
6	Bladder	During surgery	TLH + LBS	Yes	Abdominal intraoperative cystorrhaphy
7	Bladder	During surgery	TLH + LRSO^7^ + LLS^8^	Yes	Intraoperative abdominal cystorrhaphy
8	Sigmoid colon	During surgery	TLH + LBS	Yes	Intraoperative laparoscopic sutures in the sigmoid colon
9	Sigmoid colon	POD 7	TLH + LBSO^9^	Yes	Laparoscopic high anterior resection and colostomy on POD 7
10	Rectum	During surgery	TLH + LLSO + LRS	Yes	Intraoperative laparoscopic sutures in the rectum
11	Rectum	During surgery	TLH + LLC^10^ + LBS	Yes	Intraoperative laparoscopic sutures in the rectum
12	Rectum	During surgery	TLH + LLSO + LRS	No	Intraoperative laparoscopic sutures in the rectum
13	Rectum	POD 3	TLH + LBS	Yes	Laparoscopic high anterior resection and colostomy on POD 3
14	Bladder and rectum	During surgery	TLH + anterior and posterior colporrhaphy	Yes	Intraoperative vaginal sutures in the rectum and cystorrhaphy

*POD* postoperative day, *TLH* total laparoscopic hysterectomy, *LBS* laparoscopic bilateral salpingectomy, *LLSO* laparoscopic left salpingo-oophorectomy, *LRC* laparoscopic right ovarian cystectomy, *LRS* laparoscopic right salpingectomy, *LRSO* laparoscopic right salpingo-oophorectomy, *LLS* laparoscopic left salpingectomy, *LBSO* laparoscopic bilateral salpingo-oophorectomy, *LLC* laparoscopic left ovarian cystectomy

**Table 2:** The result of univariate analysis and baseline characteristics of patients before and after inverse probability weighting

Valuables *n* (%)	Before inverse probability weighting				After inverse probability weighting		
	With an experienced first assistant				With an experienced first assistant		
	Yes (*N* = 1119)	No (*N* = 563)	*p* value	SMD^2^	Yes (*N* = 1119)	No (*N* = 563)	SMD
Age (y)	46.0 (43.0–49.0)	46.0 (43.0–49.0)	0.182	0.059	45.0 (42.1–50.0)	45.9 (40.2–53.0)	0.007
BMI^1^ (kg/m^2^)	22.6 (20.6–25.7)	22.4 (20.3–25.5)	0.605	0.011	22.5 (20.1–25.4)	22.5 (18.8–26.9)	< 0.001
Parous women	743 (66.4%)	367 (65.2%)	0.624	0.026	739 (66.8%)	369 (65.7%)	0.004
Comorbidity							
Hypertension	75 (6.7%)	42 (7.5%)	0.612	0.030	78 (7.0%)	38 (6.8%)	0.005
Diabetes mellitus	16 (1.4%)	25 (4.4%)	** < 0.001**	0.179	29 (2.6%)	14 (2.5%)	0.007
Prior pelvic surgery	404 (36.1%)	233 (41.4%)	**0.038**	0.109	426 (38.0%)	214 (38.1%)	0.002
Prior cesarean section	156 (13.9%)	97 (17.2%)	0.083	0.091	171 (15.3%)	86 (15.3%)	0.002
Preoperative use of a GnRH^3^ agonist	596 (53.3%)	248 (44.0%)	** < 0.001**	0.185	561 (50.1%)	282 (50.2%)	0.002
Emergent surgery	1 (0.1%)	2 (0.4%)	0.261	0.056	2 (0.2%)	1 (0.2%)	0.004
Intraoperative adhesion	102 (9.1%)	44 (8.2%)	0.409	0.047	97 (8.7%)	48 (8.5%)	0.001
Specimen weight, g	290.0 (176.5–462.4)	294.0 (188.0–463.5)	0.324	0.015	285.0 (176.8–464.1)	290.1 (184.1–472.5)	0.002
Additional operative technique							
Salpingo-oophorectomy	186 (16.6%)	97 (17.2%)	0.782	0.016	189 (16.9%)	95 (16.9%)	0.002
Diagnosis							
Pelvic organ prolapse	9 (0.8%)	4 (0.7%)	1.00	0.011	8 (0.7%)	3 (0.5%)	0.018
Endometriosis	249 (22.3%)	119 (21.1%)	0.618	0.027	245 (21.9%)	123 (21.9%)	0.001
Operation time	131.0 (102.0–168.0)	122.0 (98.0–148.5)	** < 0.001**	NA	NA	NA	NA

Values are presented as *n* (%) or median (interquartile range). According to the univariate analysis, in the group that underwent surgery with an experienced first assistant, the rates of diabetes mellitus (*p* value ≤ 0.001) and prior pelvic surgery (*p* value = 0.038) were significantly lower, and the rate of preoperative use of a GnRH agonist was significantly higher (*p* value ≤ 0.001) than in the group that underwent surgery without an experienced first assistant. The operating time was not included in the model by propensity score because it was not a pre-treatment variable. After inverse probability weighting, all 12 covariates were well balanced between the two groups (SMD less than 0.1)

*BMI* body mass index, *GnRH* gonadotropin-releasing hormone, *SMD* standardized mean difference, *NA* not assessed

After PS-IPTW, all 12 covariates were well balanced between the two groups (SMD less than 0.1), and no significant difference was observed in the incidence rate of organ injuries between the two groups (*p* = 0.348). IPTW estimator was 0.52 (95% Confidence interval [− 0.72, 1.75]) (Table 3).

**Table 3:** Univariate and Propensity score-based inverse probability of treatment weighting analyses of organ injury rate in patients who underwent laparoscopic hysterectomy for benign diseases with or without an experienced first assistant

Outcome	With an experienced first assistant	Without an experienced first assistant	IPTW^1^ estimator	*p* value
Organ injury rate (%)				
Unadjusted	0.98%	0.53%	NA^2^	0.408
PS^3^ adjusted	0.94%	0.57%	0.52 95% CI^4^ [− 0.72, 1.75]	0.348

The involvement of an inexperienced first assistant did not affect the morbidity rate of organ injuries (*p* = 0.348)

*IPTW* inverse probability of treatment weighting, *NA* not assessed, *PS* propensity score, *CI* confidence interval

## Discussion

In this study, we evaluated the impact of the first assistant’s experience level on the occurrence of perioperative organ injuries. As a result, the involvement of an inexperienced first assistant in TLH for benign diseases was not found to affect the morbidity rate of organ injuries.

Regarding different surgical procedures, several studies determined the complications associated with the experience level of the first assistant. In surgeries for benign diseases, Tarik et al. reported that the training level of the first assistant in bariatric procedures did not negatively affect the occurrence of serious perioperative complications [21]. In surgeries for malignant diseases, comparable results have been reported in laparoscopic sigmoidectomies and laparoscopic hepatectomies [14, 22].

During TLH, the first assistant has several important roles. The skills of the first assistant are an essential part of an effective surgical team because first assistants serve as an integral bridge between primary surgeons and patients [23]. Above all, the surgical camera plays a vital role in the success of any laparoscopic procedure. Techniques that ensure optimal visualization are imperative for first assistants [24]. Moreover, they must identify the dissection plane, grasp tissue, add adequate counter traction, and provide the appropriate surgical field view for primary surgeons [25]. First assistants also provide suitable guidance and second opinions intraoperatively if needed [26, 27].

A primary surgeon may experience stress when working with an inexperienced assistant [14]. Cai et al. described potential reasons why surgery with an inexperienced assistant does not affect the morbidity of complications [22]. First, the lack of experience of the first assistant surgeon could be offset by the rest of the surgical team. If the primary surgeon knows that the first assistant surgeon is less experienced, he or she may consciously verbalize all instructions more clearly to enable better support by the first assistant surgeon. The primary surgeon may also involve more assistants in the surgery for a more even division of tasks. Second, appropriate selection of the surgical assistant based on his or her experience level may occur in consideration of case difficulty and the primary surgeon’s experience. In our study, the group of experienced first assistants had a slightly longer operative time than the group that did not. It may be that experienced first assistants were selected for more difficult cases. Furthermore, Igor et al. stated that primary surgeons may alter their technique based on the experience level of assistant surgeons to ensure surgical safety [27].

The median BMI of the operated women was approximately 22. Compared to patients with normal BMI, the high BMI patients who underwent TLH reported no particular increase in complications [28], which would be unlikely to account for the relatively low rate of organ damage in this study.

One strength of this study is that it included detailed perioperative information from our surgical database. Moreover, all patients received standardized and homogeneous management at our hospital. On the contrary, this study also has several limitations, and the results should be interpreted with caution. First, this study has inherent bias associated with operative factors that may not be referenced from the medical records (e.g., the degree of surgical difficulty and how to handle the organization by the surgeon). Second, our study reflected the experience of a single hospital, and the findings may not be generalizable to other institutions and settings. Lastly, TLH has low rates of perioperative complications and is considered relatively safe. Therefore, it may be difficult to detect significant differences in the rates of perioperative complications between patients in this cohort.

## Conclusion

In a population of women who underwent TLH performed by experienced primary surgeons for benign diseases at Teine Keijinkai Hospital, the involvement of an inexperienced first assistant did not affect the morbidity rate of organ injuries. Considering the experience level of the first assistant and the difficulty of surgical cases, the participation of inexperienced first assistants in TLH should, therefore, be encouraged for training and exposure.

## Supplementary Information

Below is the link to the electronic supplementary material.

Supplementary file1 (PDF 83 KB)

Supplementary file2 (TXT 328 KB)
