# Supplementary material for: Does the experience of the first assistant affect organ injuries in laparoscopic hysterectomy for benign diseases?
Source: Arch Gynecol Obstet. 2022 Sep 1;307(2):453–8. doi: 10.1007/s00404-022-06745-4 (PMC9918563; doi:10.1007/s00404-022-06745-4)
Supplement: Supplementary file 1 — Supplementary file1 (PDF 83 KB) [file 404_2022_6745_MOESM1_ESM.pdf]

Table S1:

The clinical path for TLH[1] at Teine Keijinkai Hospital.

Patients are admitted one day before surgery, and they see an anesthesiologist on the same day. They receive appropriate antibiotics within 60 minutes after an incision is made in the operating room. Intraoperative redosing is performed every three hours. Patients undergo a blood test and a medical examination that is performed by an attending physician on POD[2] 2 and are discharged on POD 3.

Abbreviations:

1. TLH, total laparoscopic hysterectomy
2. POD, postoperative day

Table S2:

Definitions and methods of measured variables.

Variable	Definition	Measurement method
Parous woman	A woman who has given birth to one or more babies	Medical examination conducted through interview
Prior pelvic surgery	A history of pelvic surgery	
Prior cesarean section	A history of cesarean section	
Preoperative use of a gonadotropin-releasing hormone agonist (GnRH[1] agonist)	A GnRH agonist is used to lower sex hormone levels and reduce the size of the uterus	Consulted the medical record
Emergent surgery	A surgery that is not preliminarily planned	
Specimen weight, g	Weight of the total retrieved specimen	
Surgeon type		
Experienced operator	A surgeon certified by the Skill Qualification Committee of the Japan Society of Gynecologic and Obstetric Endoscopy and Minimally Invasive Therapy or a surgeon with equivalent surgical skills	Consulted the operative note
Experienced assistant	An assistant certified by the Skill Qualification Committee of the Japan Society of Gynecologic and Obstetric Endoscopy and Minimally Invasive Therapy or a surgeon with equivalent surgical skills	

Additional operative technique	
Salpingo-oophorectomy	Resection of the ovaries and fallopian tubes
Diagnosis	
Pelvic organ prolapse	Drooping of the uterus, bladder, rectum, or vagina that allows escape from the vagina
Endometriosis	The presence and growth of functioning endometrial tissue in places other than the uterus

Abbreviations:

1. GnRH, gonadotropin-releasing hormone
